# D-galactose-induced aging aggravates obesity-induced bone dyshomeostasis

**DOI:** 10.1038/s41598-022-12206-4

**Published:** 2022-05-20

**Authors:** Napatsorn Imerb, Chanisa Thonusin, Wasana Pratchayasakul, Busarin Arunsak, Wichwara Nawara, Benjamin Ongnok, Ratchaneevan Aeimlapa, Narattaphol Charoenphandhu, Nipon Chattipakorn, Siriporn C. Chattipakorn

**Affiliations:** 1 Neurophysiology Unit, Cardiac Electrophysiology Research and Training Center, Faculty of Medicine, Chiang Mai University, Chiang Mai, 50200 Thailand; 2 Center of Excellence in Cardiac Electrophysiology, Chiang Mai University, Chiang Mai, Thailand; 3 Department of Oral Surgery, Faculty of Dentistry, Chiang Mai University, Chiang Mai, Thailand; 4 Department of Physiology, Faculty of Medicine, Chiang Mai University, Chiang Mai, Thailand; 5 Department of Physiology, Faculty of Science, Mahidol University, Bangkok, Thailand; 6 Center of Calcium and Bone Research (COCAB), Faculty of Science, Mahidol University, Bangkok, Thailand; 7 Institute of Molecular Biosciences, Mahidol University, Salaya, Nakhon Pathom Thailand; 8 The Academy of Science, The Royal Society of Thailand, Bangkok, Thailand; 9 Department of Oral Biology and Diagnostic Sciences, Faculty of Dentistry, Chiang Mai University, Chiang Mai, Thailand

**Keywords:** Physiology, Metabolic disorders, Nutrition disorders

## Abstract

We aimed to compare the time-course effect of D-galactose (D-gal)-induced aging, obesity, and their combined effects on bone homeostasis. Male Wistar rats were fed with either a normal diet (ND; n = 24) or a high-fat diet (HFD; n = 24) for 12 weeks. All rats were then injected with either vehicle or 150 mg/kg/day of D-gal for 4 or 8 weeks. Blood was collected to measure metabolic, aging, oxidative stress, and bone turnover parameters. Bone oxidative stress and inflammatory markers, as well as bone histomorphometry were also evaluated. Additionally, RAW 264.7 cells were incubated with either D-gal, insulin, or D-gal plus insulin to identify osteoclast differentiation capacity under the stimulation of receptor activator of nuclear factor κB ligand. At week 4, D-gal-induced aging significantly elevated serum malondialdehyde level and decreased trabecular thickness in ND- and HFD-fed rats, when compared to the control group. At week 8, D-gal-induced aging further elevated advanced glycation end products, increased bone inflammation and resorption, and significantly impaired bone microarchitecture in HFD-fed rats. The osteoclast number in vitro were increased in the D-gal, insulin, and combined groups to a similar extent. These findings suggest that aging aggravates bone dyshomeostasis in the obese condition in a time-dependent manner.

## Introduction

Bone plays an important role in physical movement. Unfortunately, the aging process leads to an imbalance between bone formation and bone resorption, resulting in osteoporosis and consequently increased risk of fracture^1^. Several evidences have shown that obesity exerts the effects that were associated with all nine hallmarks of aging, including genomic instability, telomere attrition, epigenetic alterations, loss of proteostasis, deregulated nutrient sensing, mitochondrial dysfunction, cellular senescence, stem cell exhaustion, and changes in intercellular communication^2,3^. Hence, obesity has been identified as a condition that mimics aging, which may provide undesired synergistic reciprocity on the aging phenomenon^3^. In addition, adipose tissue dysfunction in cases of obesity affects bone integrity via the stimulation of various inflammatory adipokines, as well as a reduction of an anti-inflammatory adipokine: adiponectin, and a bone-derived hormone: osteocalcin^4^. As a result, an association between obesity and increased bone loss has been widely observed^5,6^.

D-galactose (D-gal) is a monosaccharide sugar that is a basic substrate for the biosynthesis of many macromolecules in the body before being metabolized into glucose^7^. Chronic administration of D-gal results in a metabolic shift into undesirable metabolic pathways^8,9^. This is due to an increase in harmful substances, such as advanced glycation end products (AGEs), receptors for advanced glycation end products (RAGE) and nicotinamide adenine dinucleotide phosphate (NADPH) oxidase^8,9^. All of which cause excessive accumulation of reactive oxygen species (ROS) and mitochondrial damage^10,11^. In other words, prolonged D-gal administration leads to pathological alterations that exhibited significant similarities with the natural aging model, which was also evidenced by the increases in p16, p21 protein expressions and SA-β-gal positive cells^12–14^.

Although it is anticipated that the incidence of obesity in aging will escalate over the next decade^15^, the negative impact of aging in association with obesity on bone health has never been compared with either aging or the obesity alone. Therefore, this study aimed to compare the time-course effect of aging, obesity, and the aged-obese condition on bone homeostasis, by using D-gal and a high-fat diet (HFD) to induce aging and obese-insulin resistance, respectively, in rats.

## Results

### D-galactose administration and HFD consumption cause peripheral insulin resistance to an equal extent. However, only HFD consumption leads to dyslipidemia

Details regarding food intake, anthropometry, and metabolic parameters are shown in Table 1. Food intake was no different between groups at both week 4 and week 8. At both timepoints, body weight and visceral fat weight were heavier in rats fed with a HFD than those on a normal diet (ND), both with and without D-gal injection. Fasting glucose levels were no different between groups at both timepoints. Plasma insulin level, Homeostasis Model Assessment (HOMA-IR), and area under the curve (AUC) of glucose from 2-h oral glucose tolerance test (OGTT) were greater in the following groups: normal diet with D-gal (NDD), high-fat diet with vehicle (HFV), and high-fat diet with D-gal (HFDD) at both week 4 and week 8, when compared with the normal diet with vehicle (NDV) rat group (Table 1). With regard to groups with or without D-gal administration, total cholesterol and LDL levels were higher, but HDL level was lower in HFD-fed rats, when compared with ND-fed rats (Table 1). However, there were no changes in triglyceride levels between all groups at both timepoints (Table 1). Interestingly, D-gal administration did not aggravate the metabolic disturbance in HFD-fed rats. These findings suggest that D-gal and HFD-induced obesity increased peripheral insulin resistance to an equal extent, but only HFD-induced obesity caused dyslipidemia.

**Table 1:** Food intake, anthropometry, and metabolic parameters from an in vivo study.

Parameters	Week 4				Week 8			
	NDV	NDD	HFV	HFDD	NDV	NDD	HFV	HFDD
Food intake (g/day)	23.55 ± 1.1	22.98 ± 1.3	24.38 ± 0.8	23.42 ± 0.9	24.46 ± 1.1	23.74 ± 1.2	23.88 ± 0.8	24.53 ± 1.0
Body weight (g)	460 ± 11.5	472.7 ± 13.3	635.8 ± 6.5*^†^	638.9 ± 9.6*^†^	473.6 ± 8.4	479.7 ± 7.6	645.3 ± 8.7*^†^	655.7 ± 15.2*^†^
Visceral fat weight (g)	19.75 ± 1.5	23.58 ± 1.1	47.26 ± 4.9*^†^	54.84 ± 4.2*^†^	22.86 ± 1.1	23.91 ± 1.2	54.98 ± 1.8*^†^	60.87 ± 2.5*^†^
Plasma glucose (mg/dL)	130.4 ± 4.7	128.9 ± 6.1	127.9 ± 5.08	129.7 ± 4.6	144.2 ± 9.1	141.9 ± 10.8	147.5 ± 12.4	145.8 ± 9.8
Plasma insulin (ng/ml)	4.26 ± 0.6	9.856 ± 1.9*	8.956 ± 0.9*	12.01 ± 1.4*	4.459 ± 0.9	11.34 ± 3.05*	10.57 ± 2.2*	12.84 ± 2.2*
AUC of glucose (mg/dl × min × 10^4^)	2.34 ± 0.1	3.16 ± 0.1*	3.06 ± 0.3*	2.98 ± 0.8*	2.11 ± 0.2	3.07 ± 0.2*	3.09 ± 0.3*	2.77 ± 0.1*
HOMA-IR	19.28 ± 2.0	56.98 ± 4.2*	71.93 ± 4.1*	74.84 ± 9.2*	20.49 ± 2.2	97.17 ± 11.4*	93.20 ± 9.9*	79.13 ± 14.2*
Triglycerides (mg/dL)	89.87 ± 5.4	89.59 ± 2.9	87.04 ± 7.2	99.58 ± 1.6	86.82 ± 5.6	89.06 ± 3.1	84.42 ± 4.8	84.11 ± 3.8
Total cholesterol (mg/dL)	81.19 ± 4.0	84.11 ± 2.7	109.5 ± 14.9*^†^	105.7 ± 5.2*^†^	78.77 ± 5.7	80.35 ± 2.6	122.8 ± 4.9*^†^	112.1 ± 3.9*^†^
HDL cholesterol (mg/dL)	34.45 ± 1.3	32.7 ± 1.0	24.33 ± 1.0*^†^	24.72 ± 1.6*^†^	35.31 ± 0.9	33.32 ± 1.5	25.27 ± 2.5*^†^	24.0 ± 3.5*^†^
LDL cholesterol (mg/dL)	23.69 ± 3.5	25.77 ± 3.1	44.11 ± 5.0*^†^	42.32 ± 4.0*^†^	25.73 ± 2.1	26.59 ± 2.2	44.90 ± 4.4*^†^	48.13 ± 1.6*^†^

Data are reported as mean ± SEM (n = 6/group).

*p < 0.05 versus NDV at the same time point.

^†^*p* < 0.05 versus NDD at the same time point.

^‡^*p* < 0.05 versus HFV at the same time point.

^#^*p* < 0.05 versus same group at the different time point.

### D-galactose administration and obesity led to increased systemic aging in a time-dependent manner, and D-galactose and obesity have synergistic effects on increased systemic aging

**Figure 1:**
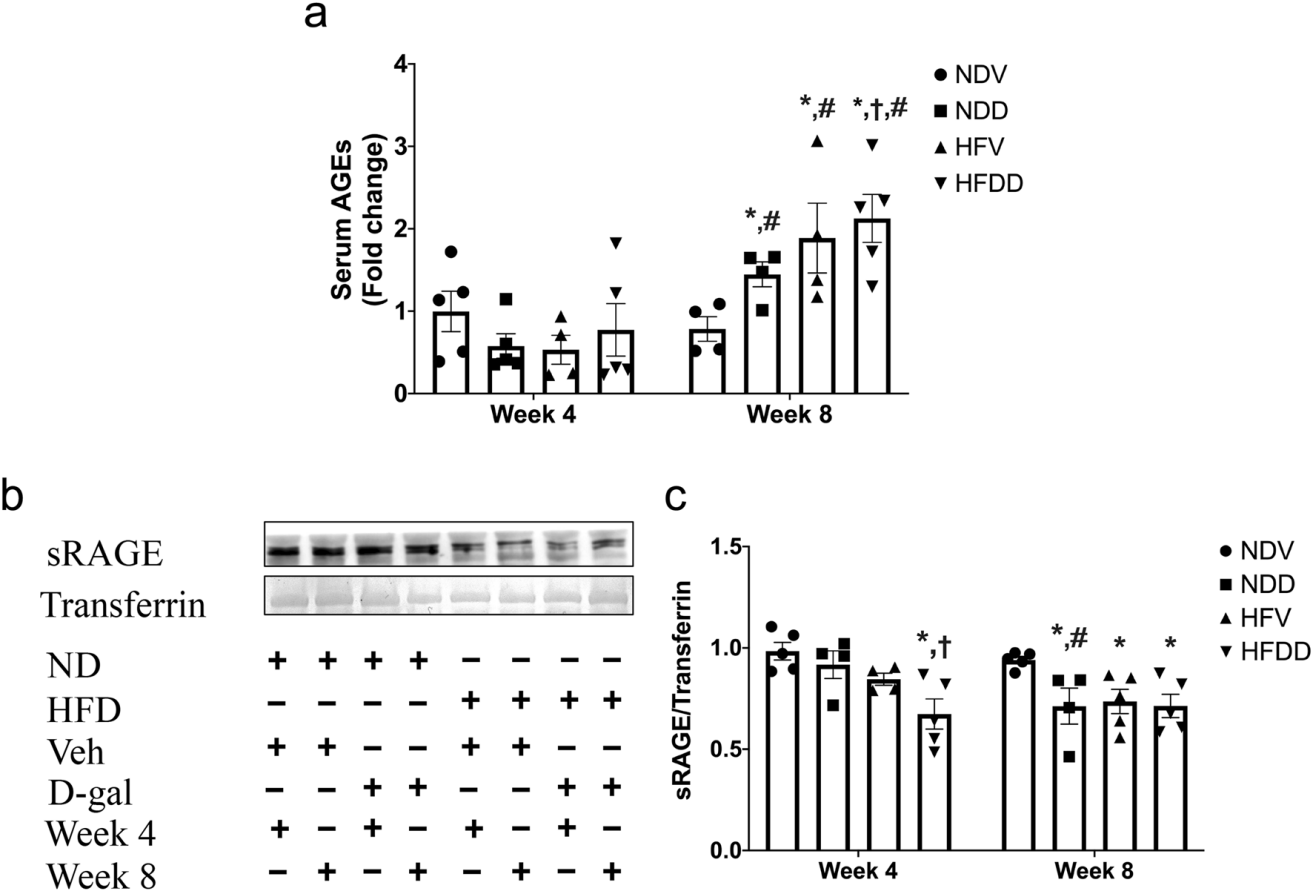
D-galactose-induced aging aggravated aging-related biomarkers in the obese-insulin resistant condition. (**a**) Quantification of serum AGEs level in NDV, NDD, HFV, HFDD at 4-week and 8-week timepoints, (**b**, **c**) sRAGE protein expression relative to transferrin as analyzed by western blotting. Bar graphs presented as mean ± SEM (n = 4–6/group). * *p* < 0.05 versus NDV at the same time point, † p < 0.05 versus NDD at the same time point, ‡ *p* < 0.05 versus HFV at the same time point, # *p* < 0.05 versus same group at the different time point. AGEs, advanced glycation end products; HFDD, high-fat diet with D-galactose; HFV, high-fat diet with vehicle; NDD, normal diet with D-galactose; NDV, normal diet with vehicle; sRAGE, soluble receptor for advanced glycation end products.

The aging process was determined via the measurement of serum AGEs and soluble receptor for advanced glycation end products (sRAGE). Deposition of AGEs has reportedly shown a correlation with osteoporosis^16^. Serum AGEs levels were not significantly different between groups at week 4. However, level of AGEs of NDD, HFV, and HFDD groups at week 8 were significantly increased, when compared to that of the NDV group and the same treatment group at week 4 (Fig. 1a). Interestingly at week 8, serum AGEs level of the HFDD group was greater than that of the NDD group (Fig. 1a). Since a soluble form of RAGE (sRAGE) plays an important role as a decoy to eliminate circulating AGEs^16^, sRAGE is normally protective against the AGEs-RAGE complex reaction, thereby preventing tissue damage and dysfunction^16^. We observed that serum sRAGE level of the HFDD group at week 4 was lower than that of the NDV, NDD and HFV groups. At week 8, sRAGE level of NDD, HFV and HFDD groups was lower than that of the NDV group (Fig. 1b, c). Taken together, our findings suggested that D-gal injection and obesity increased circulating aging to an equal extent in a time-dependent manner, and also that there is a synergistic effect of D-gal and obesity on systemic aging.

### D-galactose-induced aging aggravates obesity-induced systemic oxidative stress in a time-dependent manner

**Figure 2:**
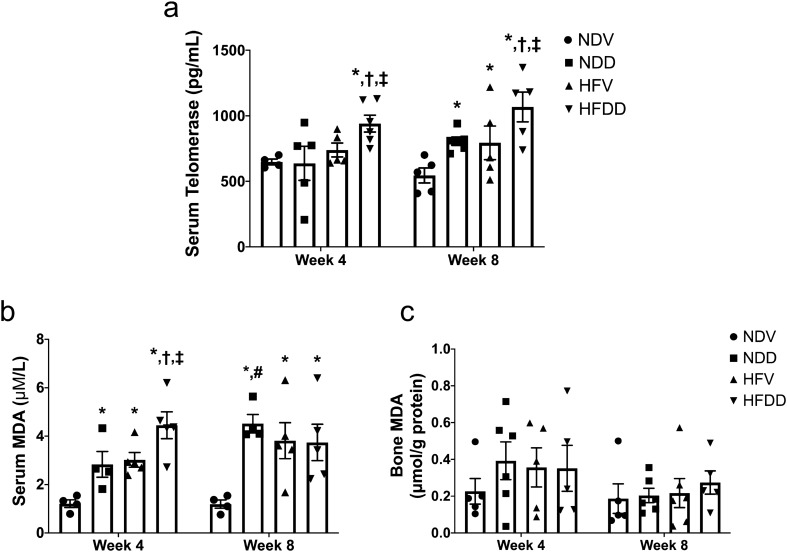
D-galactose-induced aging aggravated systemic oxidative stress in the obese-insulin resistant condition. (**a**) Levels of telomerase in serum at the indicated time points, (**b**) Levels of MDA concentration in serum at the indicated time points, (**c**) Levels of MDA concentration in skeletal tissue at the indicated time points. Bar graphs presented as mean ± SEM (n = 4–6/group). * *p* < 0.05 versus NDV at the same time point, † p < 0.05 versus NDD at the same time point, ‡ *p* < 0.05 versus HFV at the same time point, # *p* < 0.05 versus same group at the different time point. HFDD, high-fat diet with D-galactose; HFV, high-fat diet with vehicle; MDA, Malondialdehyde; NDD, normal diet with D-galactose; NDV, normal diet with vehicle.

An imbalance between oxidative metabolism and redox capacity is one of the crucial factors which contributes to aging- and obesity-related pathological conditions^17,18^. In this study, serum telomerase level and serum/ bone MDA (Malondialdehyde) level were measured to discover the severity of oxidative stress. At week 4, serum telomerase level of the HFDD group was higher, when compared to that of the other groups (Fig. 2a). At week 8, serum telomerase level of NDD, HFV and HFDD groups had become greater than that of the NDV group (Fig. 2a). Furthermore, serum telomerase level of the HFDD group at week 8 was higher than that of the NDD and HFV groups (Fig. 2a). With regard to serum MDA level at week 4 and week 8, NDD, HFV, and HFDD groups had higher levels than that of the NDV group at the same timepoint. Interestingly, the HFDD group also exhibited higher serum MDA level at week 4 than that of NDD and HFV groups (Fig. 2b). Nevertheless, bone MDA level did not differ between groups (Fig. 2c). These findings indicated that D-gal aggravated an increase in systemic oxidative stress in HFD-fed rats in a time-dependent manner.

### D-galactose-induced aging aggravates obesity-induced bone inflammation in a time-dependent manner

**Figure 3:**
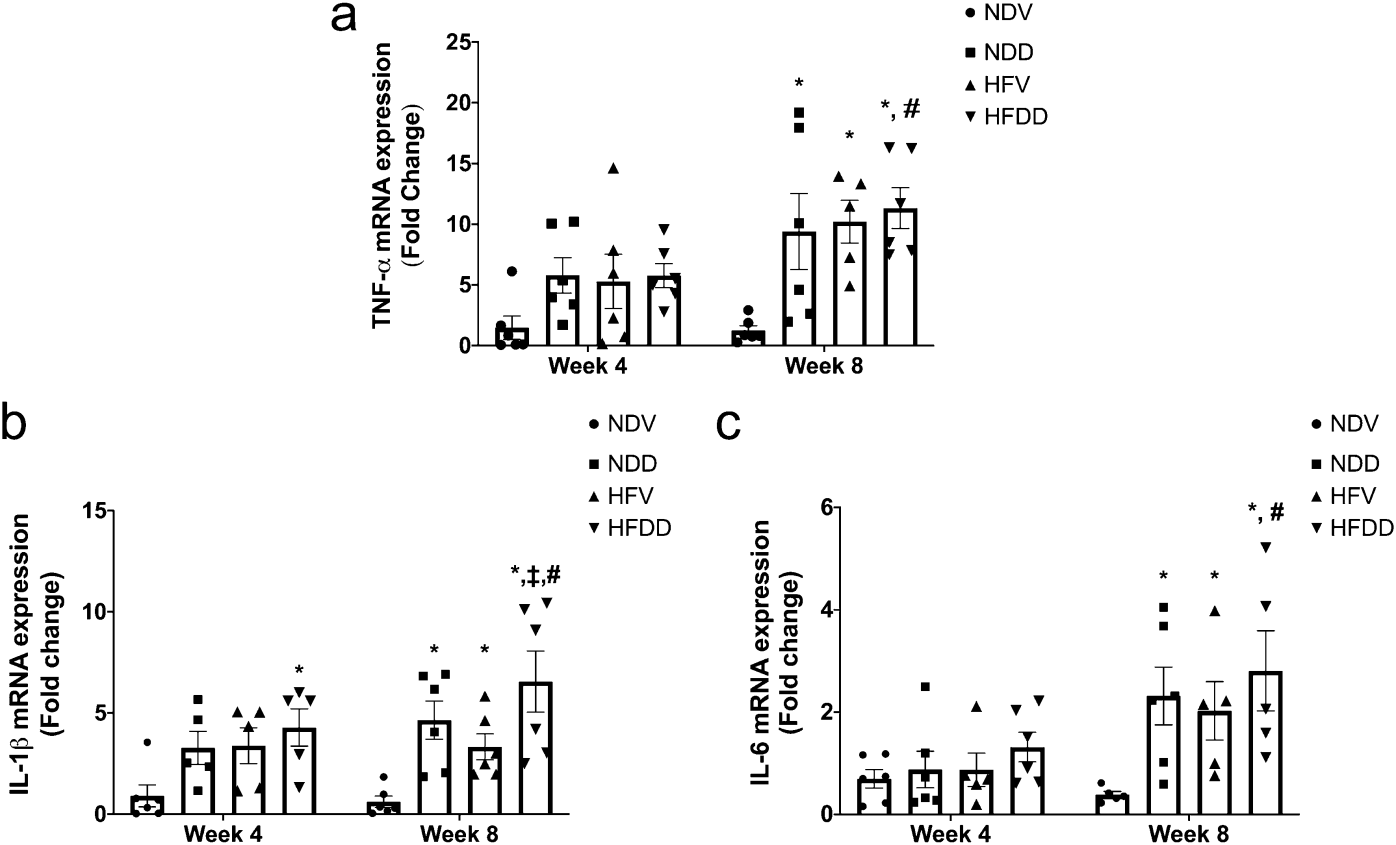
D-galactose-induced aging aggravated bone inflammation in the obese-insulin resistant condition. (**a**) TNF-α mRNA expression in bone tissues at the indicated time points, (**b**) IL-1 β mRNA expression in bone tissues at the indicated time points, (**c**) IL-6 mRNA expression in bone tissues at the indicated time points. Bar graphs are presented as mean ± SEM (n = 5–6/group). * *p* < 0.05 versus NDV at the same time point, † *p* < 0.05 versus NDD at the same time point, ‡ *p* < 0.05 versus HFV at the same time point, # *p* < 0.05 versus same group at the different time point. HFDD, high-fat diet with D-galactose; HFV, high-fat diet with vehicle; IL-1 β, interleukin-1β; IL-6, interleukin-6; NDD, normal diet with D-galactose; NDV, normal diet with vehicle: TNF-α, Tumor necrosis factor alpha.

Inflammation is a critical pathologic factor contributing to aging- and obesity-associated bone metabolic diseases^19^. Therefore, we performed TNF-α, IL-1β, and IL-6 mRNA expression analysis in the bone, in order to investigate the impact of D-gal-induced aging and obesity on bone inflammatory status (Fig. 3a–c). We found that the HFDD group exhibited a significantly increase in IL-1β mRNA expression at week 4, when compared to that of the NDV control (Fig. 3b). At week 8, TNF-α, IL-1β, and IL-6 mRNA expressions were remarkably elevated in NDD, HFV, and HFDD groups, when compared with those of the NDV group (Fig. 3a–c). Moreover, IL-1β mRNA expression at week 8 was significantly higher in the HFDD group than that of the HFV group, indicating the aggravating effect of D-gal-induced aging on increased bone inflammation in obesity (Fig. 3b). Interestingly, TNF-α, IL-1β, and IL-6 mRNA expressions were significantly higher in HFDD at week 8, as compared with the same group at week 4 timepoint (Fig. 3a–c). All of these findings suggested that D-gal-induced aging aggravates obesity-induced bone inflammation in a time-dependent manner.

### D-galactose-induced aging aggravates obesity-induced osteoclastogenesis and bone resorption in a time-dependent manner

**Figure 4:**
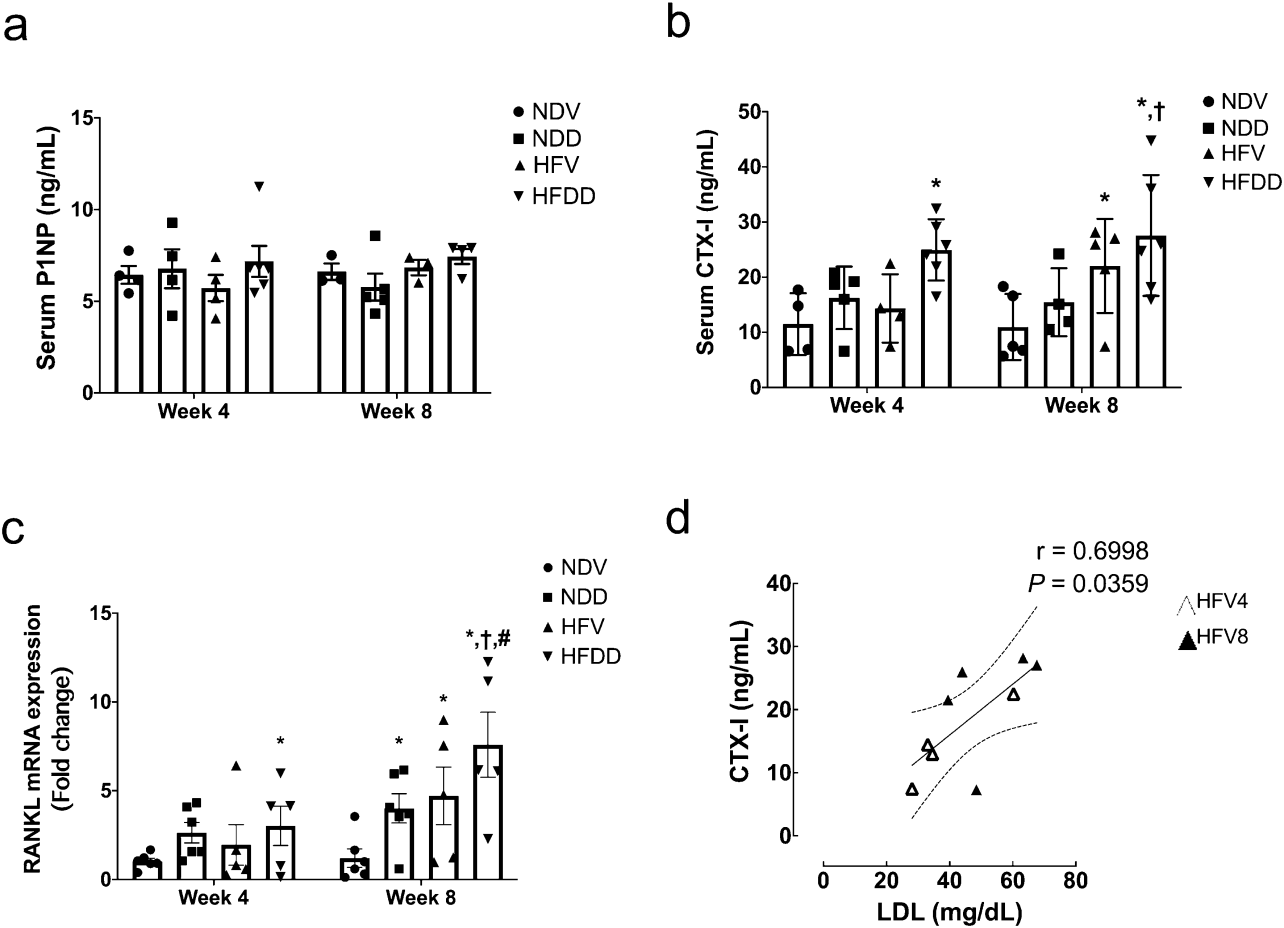
D-galactose-induced aging aggravated bone resorption markers in the obese-insulin resistant condition. (**a**) Levels of P1NP in serum at the indicated time points, (**b**) Levels of CTX-I in serum at the indicated time points. Bar graphs are presented as mean ± SEM (n = 4–6/ group). (**c**) RANKL mRNA expression in bone tissues at the indicated time points. (**d**) Correlation between CTX-I and LDL. * *p* < 0.05 versus NDV at the same time point, † *p* < 0.05 versus NDD at the same time point**,** ‡ *p* < 0.05 versus HFV at the same time point**,** # *p* < 0.05 versus same group at the different time point. CTX-I, C-terminal telopeptide of type I collagen; HFDD, high-fat diet with D-galactose; HFV, high-fat diet with vehicle; NDD, normal diet with D-galactose; NDV, normal diet with vehicle; P1NP, procollagen type I N-terminal propeptide; RANKL, receptor activator of nuclear factor κB ligand.

To determine bone homeostasis, we investigated the levels of bone formation and resorption markers in serum, which were procollagen type I N-terminal propeptide (P1NP) and C-terminal telopeptide of type I collagen (CTX-I), respectively. Moreover, RANKL mRNA expression as a marker of osteoclastogenesis was evaluated in the bone. We found that serum P1NP level was unaltered in all groups at both timepoints (Fig. 4a). At week 4, serum CTX-I level and RANKL mRNA expression in the bone were significantly higher in the HFDD group, when compared to those of the NDV group. At week 8, serum CTX-I level was greater in HFV and HFDD groups than that of the NDV group (Fig. 4b). Consistently, RANKL mRNA expression at week 8 was significantly elevated in NDD, HFV, and HFDD group, as compared with that of the NDV group (Fig. 4c). Additionally, in comparison with the NDD group at the same timepoint, higher levels of serum CTX-I and RANKL mRNA expression in the bone of the HFDD group were observed at week 8 (Fig. 4b, c). Interestingly, RANKL mRNA expression in the bone of the HFDD group was significantly higher at week 8, as compared to the same group at week 4 timepoint (Fig. 4c). Correlation studies revealed the positive correlation between circulating CTX-I and serum LDL (r=0.3952, *P*=0.0079; Supplementary Fig. 1a), particularly in HFV4 and HFV8 (r=0.6998, *P*=0.0359; Fig. 4d). Together, these findings suggested that D-gal-induced aging aggravates obesity-induced bone resorption in a time-dependent manner.

### D-galactose exposure and hyperinsulinism independently stimulate osteoclast differentiation

**Figure 5:**
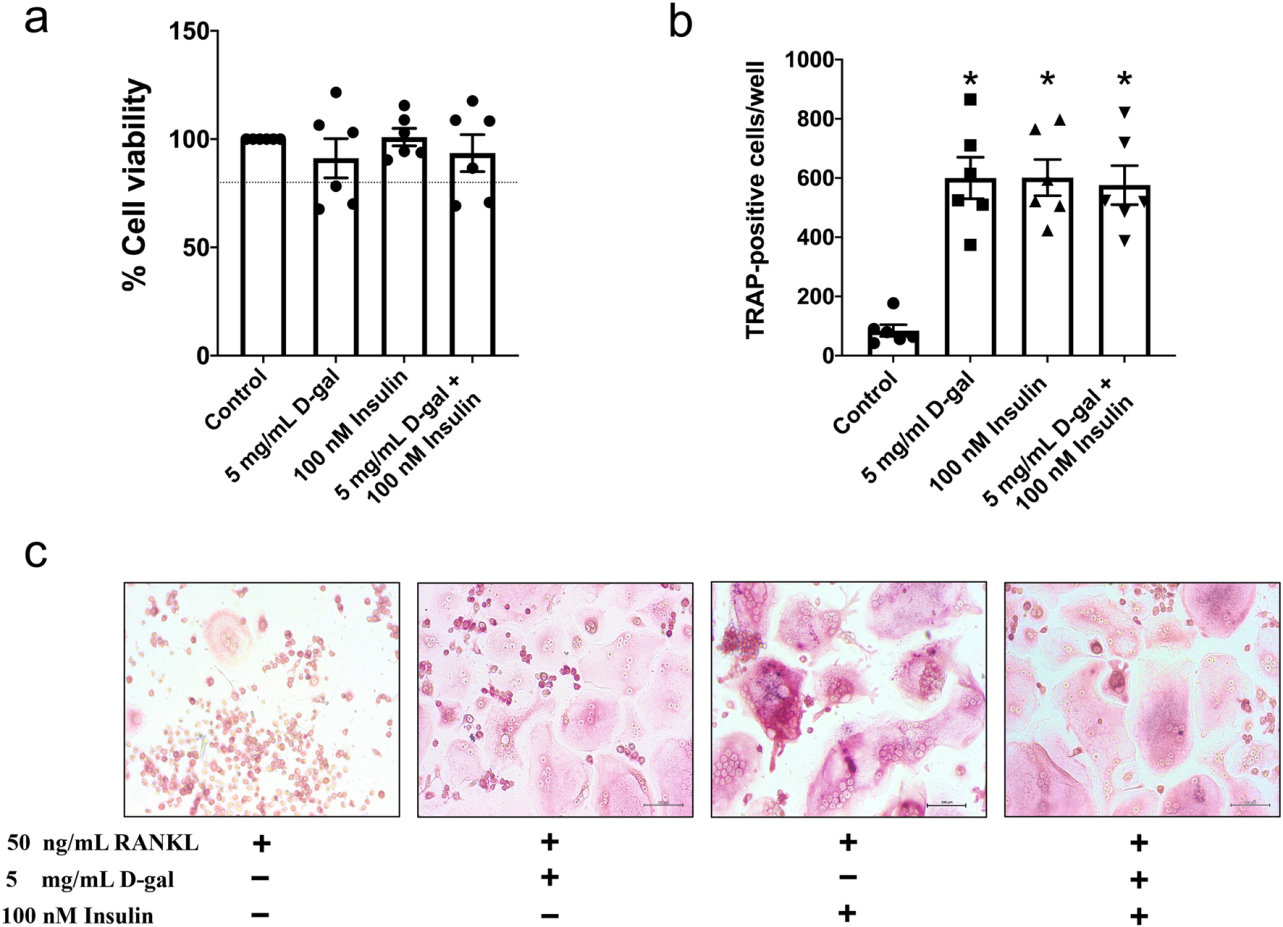
D-galactose-induced senescence and hyperinsulinism-stimulated insulin resistance accelerated RANKL-induced osteoclast differentiation in RAW 264.7 cells. (**a**) % cell viability of RAW 264.7 belonging to four treatment group, (**b**) Number of TRAP-positive multinucleated cells which presented more than three nuclei indicated a mature osteoclast. (**c**) TRAP positive staining of RANKL-stimulated multinucleated osteoclasts with the treatments of control, 5 mg/mL D-galactose, 100 nM insulin and the combined treatment (20 × magnification). Bar graphs presented as mean ± SEM (n = 6/group).* *p* < 0.05 versus control group. D-gal, D-galactose; RANKL, receptor activator of nuclear factor κB ligand; TRAP, tartrate-resistant acid phosphatase.

As previously mentioned, an increased serum CTX-I level was present in the rats after either D-gal- or HFD-induced hyperinsulinemia. We sought to confirm that D-gal-induced aging and the insulin-resistant condition contributed to an increase in bone resorption by increasing the number of osteoclasts. Therefore, we went on to conduct an *in vitro* experiment to evaluate the capacity of osteoclast differentiation following D-gal exposure alone, hyperinsulinism alone, and a combination of D-gal exposure plus hyperinsulinism, using RAW 264.7 cells. The results showed that D-gal at a dose of 5 mg/mL and insulin at a dose of 100 nM could maintain cell viability of more than 80% (Fig. 5a). In comparison to the control, the number of TRAP-positive multinucleated osteoclasts significantly increased in the cells treated with D-gal alone, insulin alone, and D-gal plus insulin (Fig. 5b, c). These results supported the findings that D-gal- and obesity-induced bone loss were observed in our *in vivo* experiment. Surprisingly, the combined treatment with D-gal and insulin did not further increase osteoclast differentiation when compared with the treatment with D-gal or insulin alone.

### D-galactose-induced aging aggravates obesity-induced bone loss and impaired bone architecture in a time-dependent manner

**Figure 6:**
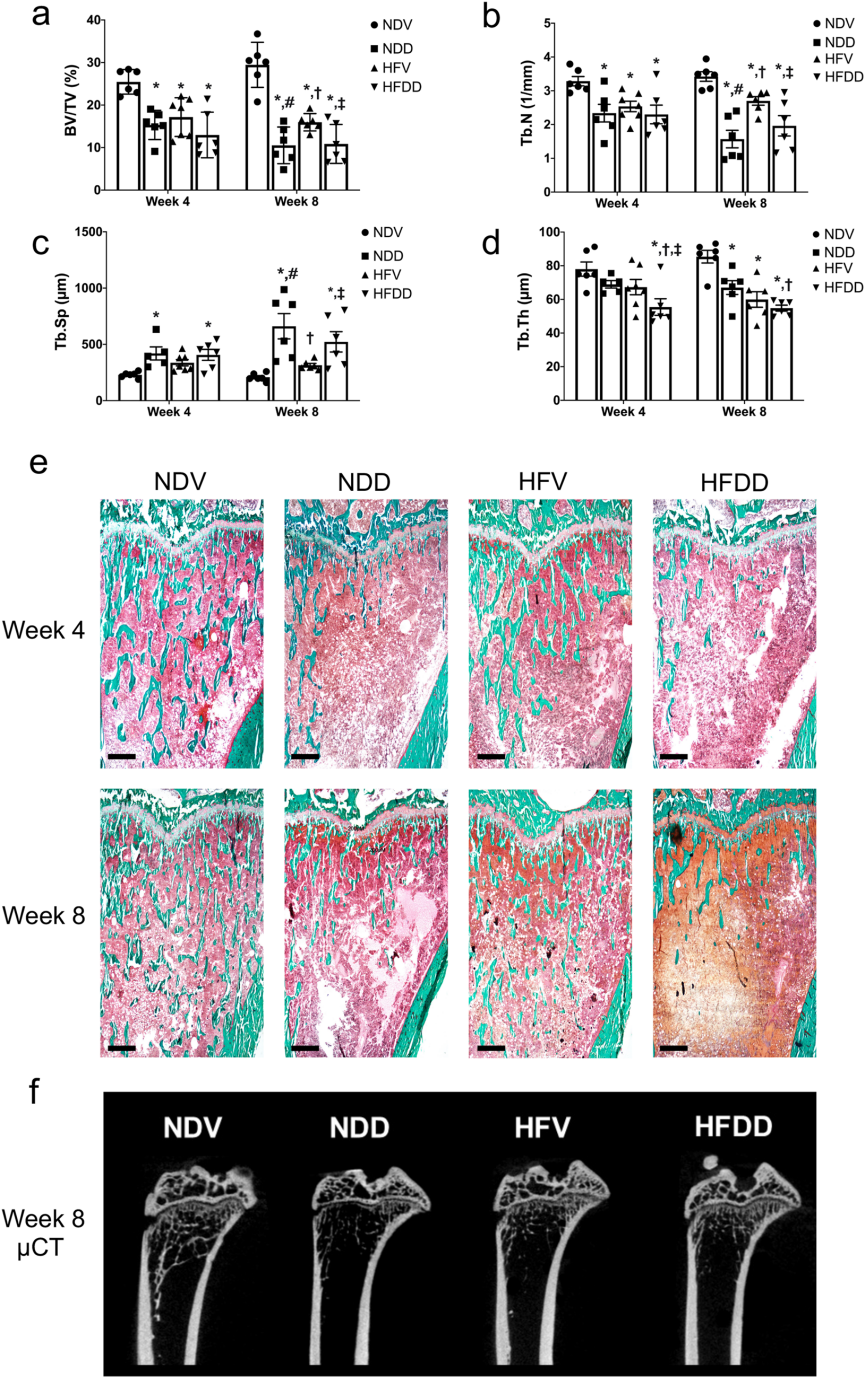
Synergistic effect of D-galactose-induced aging in obese-insulin resistance influences the impairment of bone microarchitecture and severe bone volume loss. (**a**) Quantification of trabecular bone volume per tissue volume (BV/TV) at the indicated time points, (**b**) Quantification of trabecular number (Tb.N) at the indicated time points, (**c**) Levels of trabecular separation (Tb.Sp) at the indicated time points, (**d**) Levels of trabecular thickness (Tb.Th) at the indicated time points, (**e**) Images of right tibiae of rats with Goldner’s trichrome staining histomorphometry belonging to eight groups at indicated time points (n = 6/group). (**f**) Representative images show epiphyseal and metaphyseal regions of tibiae from 8-week 0.9% NaCl or d-galactose-injected rats. The images were obtained from ultra-high-resolution microcomputed tomography (μCT). Scale bars = 1000 µM (× 20 magnification). Bar graphs presented as mean ± SEM. * *p* < 0.05 versus NDV at the same time point, † *p* < 0.05 versus NDD at the same time point, ‡ *p* < 0.05 versus HFV at the same time point, # *p* < 0.05 versus same group at the different time point. BV/TV, bone volume per tissue volume; HFDD, high-fat diet with D-galactose; HFV, high-fat diet with vehicle; NDD, normal diet with D-galactose; NDV, normal diet with vehicle; Tb.N, trabecular number; Tb.Sp, trabecular separation; Tb.Th, trabecular thickness.

Bone histomorphometry was used to evaluate trabecular microarchitecture, including the following parameters: bone volume fraction (BV/TV), trabecular number (Tb.N), trabecular separation (Tb.Sp), and trabecular thickness (Tb.Th), as shown in Fig. 6a–e. At week 4, BV/TV and Tb.N in the NDD, HFV, and HFDD groups were lower than those of the NDV group (Fig. 6a, b). With regard to Tb.Sp, its value at week 4 was greater in the NDD and HFDD groups, when compared with the NDV group (Fig. 6c). Interestingly, Tb.Th at week 4 of the HFDD group was lower that of the NDV, NDD and HFV groups (Fig. 6d). Consistent with the findings at week 4, BV/TV and Tb.N at week 8 were found to be lower in NDD, HFV, and HFDD groups than in those of the NDV group. Furthermore, BV/TV and Tb.N of NDD and HFDD groups were lower, when compared to that of the HFV group (Fig. 6a, b). Tb.Sp at week 8 was higher in the NDD and HFDD groups than in the NDV and HFV groups. (Fig. 6c). However, at week 8, Tb.Th was lower in NDD, HFV, and HFDD groups, when compared with that of NDV group. The Tb.Th value at week 8 of the HFDD group was also lower than that of the NDD group at the same timepoint (Fig. 6d). The results from ultra-high resolution µCT confirmed the findings obtained from bone histomorphometry (Fig. 6f). All of these findings suggested that D-gal-induced aging leads to worsening obesity-induced bone loss and disrupted bone architecture in a time-dependent manner.

## Discussion

Both *in vivo* and *in vitro* studies were used to identify the effects of D-gal-induced aging, obesity, and D-gal plus HFD-induced aging and obesity on metabolic parameters, systemic aging, systemic oxidative stress, systemic inflammation, and bone homeostasis. Our major findings are summarized in Table 2.

**Table 2:** A summary of all findings. D-galactose-induced aging aggravated obesity-induced systemic oxidative stress, bone inflammation, and bone dyshomeostasis in a time-dependent manner. Consistent with the in vivo study findings, the in vitro study confirmed underlying mechanisms associated with severe accelerated bone loss as a consequence of D-galactose- and hyperinsulinism-stimulated osteoclastogenesis.

Wistar rats	NDV (control)		NDD		HFV		HFDD	
	Week		Week		Week		Week	
	4	8	4	8	4	8	4	8
**Age-related markers**								
Serum AGEs			↔	↑	↔	↑	↔	↑↑
Serum sRAGE			↔	↓	↔	↓	↓	↓
**Oxidative stress markers**								
Serum MDA			↑	↑↑	↑	↑↑	↑↑	↑↑
Bone MDA			↔	↔	↔	↔	↔	↔
Serum telomerase			↔	↑	↔	↑	↑	↑↑
**Inflammatory markers**								
TNF-α mRNA expression in the bone			↔	↑	↔	↑	↔	↑
IL1- β mRNA expression in the bone			↔	↑	↔	↑	↑	↑↑
IL-6 mRNA expression in the bone			↔	↑	↔	↑	↔	↑
**Bone turnover markers**								
Serum P1NP			↔	↔	↔	↔	↔	↔
Serum CTX-I			↔	↑	↔	↑↑	↑	↑↑
RANKL mRNA expression in the bone			↔	↑	↔	↑	↑	↑↑
**Histomorphometric bone parameters**								
BV/TV			↓	↓↓	↓	↓	↓	↓↓
Tb.Th			↔	↓	↔	↓↓	↓↓	↓↓
Tb.Sp			↔	↑↑	↔	↑	↑	↑↑
Tb.N			↓	↓↓	↓	↓	↓	↓↓

RAW 264.7 cell	Control		5 mg/mL D-galactose		100 nM Insulin		5 mg/mL D-galactose + 100 nM Insulin	
Osteoclast number			↑		↑		↑	

AGEs, Advanced glycation end products; BV/TV, Bone volume per tissue volume; CTX-I, C-terminal telopeptide of type I collagen; HFD, High-fat diet; HFDD, High-fat diet with D-galactose; HFV, High-fat diet with vehicle; MDA, Malondialdehyde; NDD, Normal diet with D-galactose ; NDV, Normal diet with vehicle; P1NP, Procollagen type I N-terminal propeptide; RANKL, Receptor activator of nuclear factor κB ligand; sRAGE, Soluble receptor for advanced glycation end products; Tb.N, Trabecular number; Tb.Sp, Trabecular separation; Tb.Th, Trabecular thickness; TRAP, Tartrate-resistant acid phosphatase; ↑, Higher than NDVS group; ↑↑, Much higher than NDVS group; ↓, Lower than NDVS group; ↓↓, Much lower than NDVS group; ↔ , No statistically significant difference when compared with NDV group at the same timepoint.

We demonstrated that D-gal-induced aging and obesity resulted in equal increases in insulin resistance. However, dyslipidemia was only present in the obese condition. This finding was inconsistent with previous clinical studies, which showed that aging was a major risk factor of dyslipidemia^20,21^. However, we found an early increase in serum MDA level, a marker of systemic lipid peroxidation^22^, in rats receiving D-gal. This suggested that the rats with D-gal-induced aging also exhibited evidence of lipid metabolism dysfunction, which may later contribute to dyslipidemia. Therefore, an increment in dosage and injection period of D-gal may lead to dyslipidemia in these rats as shown in some prior studies^23,24^.

It has been observed that chronic administration of D-gal leads to the accumulation of AGEs^8,9^. The AGEs-RAGE complex reaction brings about various degenerative changes and dysfunctions^3,25,26^. This harmful reaction has been shown to cause an excessive generation of ROS and mitochondrial damage due to the lack of antioxidative capacity and an imbalance in oxidative metabolism^10,11^. These changes result in pathological alterations that mimic the aging phenomenon^12^. In our study, it was found that the effect of D-gal plus HFD-induced obesity on accelerated systemic aging was detected early through a noticeable decrease in serum sRAGE level at week 4, however serum AGEs level was not altered at this timepoint. Interestingly, an extended period of D-gal administration or HFD consumption or both caused a reduction in sRAGE levels, along with an elevation of serum AGEs at week 8. These findings suggested that our D-gal-induced aging model successfully mimicked natural aging characteristics. The serum sRAGE is a highly sensitive and early detectable aging biomarker, a later increase in the accumulation of AGEs is likely to be as a result of a diminishment of its decoy soluble receptor, sRAGE.

Although neither antioxidant-related enzymes nor antioxidative capacity was quantified in this study, serum telomerase level was investigated as an oxidative stress marker^27,28^. The effect of D-gal plus HFD-induced aging and obesity on accelerated systemic oxidative stress was detected early, at week 4, through an increase in serum telomerase level. At week 8, serum telomerase level was also increased in D-gal-induced aging alone and obesity alone. Unlike telomere length, the telomerase level was unable to determine aging^27^. For this reason, the relationships among telomerase level, telomerase activity, telomere length, AGEs, and sRAGE should be further evaluated to identify the interaction between the AGEs-RAGE complex and the telomere pathway on the development of oxidative stress and aging.

An increase in lipid peroxidation has also been recognized as being related to increased aging and obesity-associated oxidative stress^22,29,30^. In addition to higher telomerase level as mentioned above, the effect of D-gal plus HFD-induced aging and obesity on accelerated systemic oxidative stress was also detected early, at week 4, through a dramatically increased serum MDA level. Unlike serum MDA, bone MDA level did not increase significantly following any kind of treatment at either timepoint. This was inconsistent with prior studies which found higher bone MDA level in response to increased ROS production and oxidative stress in association with aging and six weeks of estrogen deprivation^31,32^. This may be explained by too short a duration of the treatment in our study, and therefore increased fat deposition in bone marrow may not be sufficiently high to exhibit significantly increased lipid peroxidation in the bone. A future study quantifying fat deposition in the bone marrow tissue is needed to explore this idea.

It has been widely accepted that excessive oxidative stress shows a strong correlation with an increase in systemic inflammation, which is a key contributor of aging- and obesity-associated metabolic bone diseases^33,34^. Specifically, excessive ROS production as a consequence of oxidative stress progressively activates the proinflammatory cascades, leading to an elevation of systemic inflammation and subsequently metabolic dysfunction^33^. Our present study demonstrated that the level of inflammatory cytokines, including TNF-α, IL-1β, and IL-6 mRNA expression were significantly higher in the bone of both ND- and HFD-fed rats after receiving 8 weeks of D-gal, when compared with those of the NDV control. These findings suggested that D-gal-induced aging and obesity extensively trigger the proinflammatory cascades, and therefore leading to increased inflammatory cytokines. Consistently, other previous studies also showed that both D-gal induced-aging rats and HFD-induced obese rats had higher levels of TNF-α, IL-1β, and IL-6 expression than those of the control group^35–37^. Taken together, all of these findings suggested that D-gal and HFD both independently and synergistically caused increased oxidative stress and bone inflammation, resulting in aging- and obesity-related bone metabolic diseases.

The negative impact of increased oxidative stress on bone turnover have been widely reported^38,39^. We observed that the D-gal plus HFD-induced aged-obese condition led to the increases in a bone resorption marker (serum CTX-I level) and RANKL mRNA expression in the bone at week 4. At week 8, D-gal plus HFD-induced aging and obesity both independently and synergistically stimulated the increases in serum CTX-I level and RANKL mRNA expression in the bone. Interestingly, the role of oxidative stress in the alteration of RANKL expression has been well-established in aging models^40,41^. Specifically, a prior study in rats revealed that 8 weeks of D-gal administration compromised the activity of antioxidant enzymes (superoxide dismutase and catalase) in serum, increased serum RANKL level, and increased osteoporosis^41^. These findings suggested that both D-gal and HFD-induced increased oxidative stress was also associated with increased RANKL expression. These was also observed in our study, as indicated by increased MDA and telomerase levels, along with increased RANKL mRNA expression in the bone. Even though previous studies demonstrated a reduction of P1NP, a bone formation marker, in both the D-gal-induced aging and obese model^42,43^, we neither observed this alteration following any kind of intervention nor at any timepoint. These contradictory results suggested that an increase in osteoclast activity was exhibited earlier than a decrease in osteoblast activity. To support this explanation, a further study extending the duration of D-gal administration is warranted.

Consistent with prior studies^5,44–46^, an impairment of trabecular bone microarchitecture following either D-gal injection or HFD consumption was discovered in our study. This structural change was detected early, at week 4, through the simultaneous loss of bone volume fraction (i.e. BV/TV) and Tb.N, suggesting that these two aspects are affected at an early stage by D-gal-induced aging and obesity. At week 8, we discovered a further deleterious effect of D-gal-induced aging and the D-gal plus HFD-induced aged-obese condition, as indicated by a marked decrease in bone volume fraction and Tb.N, as well as greater Tb.Sp. Interestingly, these alterations were barely detectable in the obese condition alone at week 8. All of the findings at week 8 supported the previous findings that obesity or an increase in body weight has a positive effect on bone and might delay the onset of osteoporosis concurrently with an increase in mechanical loading^47,48^. Bone quality is known to gradually deteriorate in obese individuals, but it takes time for the signs of impairment to become evident. We also found that the change in Tb.Th was exhibited later than any other parameters. This finding was consistent with previous studies demonstrating that Tb.Th was a bone parameter which seldom changed in response to osteoporotic progression^5,45^. Although there were significant changes in bone histomorphometric parameters (BV/TV, Tb.N and Tb,Th) after either D-gal administration or HFD consumption, the levels of CTX-I were significantly increased only in some groups of animals without any changes in P1NP levels. These findings could be explained by using a small number of animals and the end-point cross-sectional design of the study, the magnitude of the difference in the level of bone turnover markers i.e., CTX-I and P1NP can also be quite small at a specific time-point. In addition, a remarkably higher CTX-I levels in HFV8 and HFDD8 group were detected, probably due to the greater serum LDL concentrations, as compared with those of NDV and NDD group (Table 1). Specifically, we further observed that there was a positive correlation between serum LDL and CTX-I levels (Fig. 4d, Supplementary Fig. 1a, b) as similarly demonstrated in a previous study^49^. It has been known that LDL and cholesterol are able to induce the osteoclast-mediated bone resorption^50^. An extended experimental period to be more than 8 weeks and more animal number may require in a future study for demonstrating the higher magnitude of the difference in circulating bone turnover marker in which consistent to the severity of the bone defect.

It has been reported that osteoclasts are highly sensitive and susceptible to progressively increasing oxidative stress, leading to increased osteoclastogenesis and osteoblast apoptosis^51,52^. Our *in vitro* study demonstrated that D-gal and hyperinsulinism promoted osteoclast differentiation equally. Consistent with this finding an increased secretory activity of the osteoclasts has been well-established at either 8 weeks after the onset of D-gal injection- or long-term HFD consumption-induced osteoporosis, as indicated by an elevation of cathepsin K level in serum^41,53–55^. These previous findings suggested that either D-gal- or hyperinsulinism-induced increase in osteoclast number was mediated by the increased secretory activity of osteoclasts, resulting in increased osteoclast differentiation. In support of this explanation, the measurement of secretory activity of the osteoclasts, for example quantifying bALP and cathepsin K levels, should be established in a future study. However, no synergistic effects of D-gal and hyperinsulinism on osteoclast differentiation was observed in our study. This may be explained by the inadequacy of the D-gal dose and the incubation period in our study. In addition, cellular hyperinsulinism may not exactly mimic the obesity phenotype due to the lack of increased fat accumulation. Therefore, further studies using palmitic acid and a higher dose of D-gal with a longer period of incubation should be performed.

### Limitations of the study

In this study, there were no natural aging rats as a positive control. Hence, we did not know if the negative impacts of D-gal-induced aging, obesity, and their combined effects on bone homeostasis are comparable with the natural aging process and natural combined aged-obese condition-induced bone dyshomeostasis at the same age in rats. In addition, the markers of adipokines related to D-gal and HFD were not investigated in this study and the identification of changes in levels of adipokines is needed in further investigations in these models. Those findings may mediate the loss of bone mass in both aging and obese conditions. A further limitation is that our treatment duration was potentially too short, and therefore we did not see any changes in some of the bone parameters. For these reasons, a future study including naturally aging rats with a longer duration of treatment is essential in order to identify the progressive dynamic changes in bone in correlation with obesity and aging, in both natural and accelerated processes. The use of male rats only in this study could also have impacted the data as sex hormones have been identified as major regulators of the bone^56^. To address this the interaction between D-gal-induced aging, obesity, and sex hormones in bone in both male and female rats, should be investigated further. This will be helpful in determining whether the effects of D-gal-induced aging and obesity on bone are sexually dimorphic or not.

In summary, our results highlighted sequential metabolic and skeletal alterations in D-gal-induced aging, obesity, and the combined effects of aging and obesity. This study clearly demonstrated that obesity aggravated systemic aging, systemic oxidative stress, and bone dyshomeostasis in D-gal-induced aging in a time-dependent manner. Therefore, early interventions, such as caloric restriction and exercise, to alleviate the combined effect of aging and obesity as well as diabetes-related metabolic bone diseases is considered highly beneficial to reduce bone pathology, and consequently to decrease fracture risk and improve quality of life among the elderly.

## Methods

### Animals

All experimental protocols were approved by the Faculty of Medicine, Chiang Mai University Institutional Animal Care and Use Committee, in compliance with NIH guidelines (Ethic-approval number: 27/2563). All methods were carried out in accordance with relevant guidelines and regulations. All methods are also reported in accordance with ARRIVE guidelines. Six-week-old male Wistar rats (n=48) were purchased from Nomura Siam International Co, Ltd. (Bangkok, Thailand). Rats were randomly assigned to receive either a normal diet (ND) or a high-fat diet (HFD). The normal diet group (ND; n=24) were given standard laboratory chow with a total of 4.02 kcal/g and 19.77% of total energy (%E) from fat (Mouse Feed Food No. 082, C.P. Company, Bangkok, Thailand). The HFD group (HFD; n=24) were given a diet with a total of 5.35 kcal/g and 59.28% of total energy (%E) from fat, as described in a prior study^57^. At the end of week 12, both ND and HFD groups were subcutaneously injected with either 0.9% NaCl as a vehicle (NDV and HFV) or 150 mg/kg/day of D-gal to induce aging (NDD and HFDD) for 4 or 8 weeks (n=6/group). The rats were decapitated at the end of treatment (4 or 8 weeks) after being fasted for 5 hrs. Blood was then collected to for the measurement of metabolic parameters, as well as markers that are related with age, oxidative stress, and bone turnover. The right femora and tibiae were also dissected out to evaluate the MDA concentration and trabecular bone parameters, respectively. The experimental protocol is summarized in Supplementary Fig. 2.

### Determination of metabolic parameters

Colorimetric assay kits (ERBA Mannheim, Germany) were used to determine glucose, triglyceride, total cholesterol, HDL, and LDL levels. Peripheral insulin sensitivity was assessed by the quantification of plasma insulin level (sandwich ELISA kit; Millipore, MI, USA), along with HOMA-IR^5^ and the total area under the curve (AUC) of glucose from a 2-hr oral glucose tolerance test (OGTT) as described in a previous study^58^.

### Measurement of aging and bone turnover markers in serum

Aging and bone turnover markers in serum, including AGEs, P1NP, and CTX-I were measured using standard ELISA kits according to the manufacturer’s instructions. The details of each assay were described in a prior study^59^.

### Soluble receptor for advanced glycation end products (sRAGE) protein expression in serum

Serum sRAGE protein expression was determined using Western blot analysis. Each step of Western blot was detailed in a previous study^59^.

### Serum and bone malondialdehyde (MDA) Quantification

Serum and bone MDA levels were measured using high performance liquid chromatography (HPLC) as described in prior studies^59–62^.

### mRNA expression analyses in the bone

The femoral bones were homogenized for RNA isolation and submitted to quantitative real-time polymerase chain reaction (qRT-PCR) following a protocol performed in previous study^63,64^. Briefly, 1 ml of TRIzol (Invitrogen Life Technologies, Carlsbad, CA, USA) was added to the sample and homogenize using a homogenizer, before incubation at the room temperature for 10 min. All resulting solution were transferred into 1.5 ml tube. Subsequently, 200 μl of chloroform was added and vigorously mixed, allowed to stand at room temperature for 5 min before centrifugation at 12,000 rpm for 15 min at 4 °C to obtain 300-350 μl of the colorless supernatant on the upper aqueous layer. The resulting messenger ribonucleic acid (mRNA) concentration obtained from bone tissue was measured using the Take3 micro-volume plate (BioTek Instruments, Inc., USA). To obtain the purified extracted mRNA, samples with optical densities ranging between 1.8 and 2.0 determined by a 260/280 nm wavelength were used. Subsequently, single-stranded complementary DNA (cDNA) was synthesized using the iScript cDNA synthesis kit (Bio-Rad Laboratories Ltd., USA) in accordance with the manufacturer’s instructions. The qRT-PCR were conducted using the SsoFast™ EvaGreen supermix kit (Bio-Rad Laboratories Ltd., USA). The gene expressions were analyzed by qRT-PCR assay in duplication using the Bio-Rad Cx96 Detection System (Bio-Rad Laboratories Ltd., USA). The following sequence was used on each sample: 40 cycles of amplification, denaturation at 95 °C for 5 seconds, annealing at 55°C for 10 seconds and extension at 72 °C for 20 seconds. The quantification of each mRNA was normalized against the house keeping gene β-actin and quantified using the C_T_ method. The primer pairs using for RANKL, TNF-α, IL-1 β, IL-6, and β-actin used in this study are listed in Supplementary Table 1.

### Static bone histomorphometry

Bone histomorphometry was performed in the right tibiae. The details of bone preparation and analysis were described in a previous study^59,65^.

### Ultra-high-resolution microcomputed tomography (μCT)

After the removal of attached muscle and soft tissue, the tibiae were wrapped in moist gauze. An *ex vivo* scan was performed using ultra focus mode at 55 kV, 0.17 mA (E-Class VECTor^6^CT system, MILabs, Netherlands). The rotation angle was 0.25° at each step and voxel size was 10 μm^3^ isotropically. Three-dimensional bone figures were reconstructed using MILabs Rec 12.00 software.

### Cell culture

The experimental protocol of the cell culture is summarized in Supplementary Fig. 2. RAW 264.7 cell lines (third passage) were purchased from the American Type Culture Collection (ATCC® TIB-71™) and cultured in T-75 flasks with high glucose medium supplemented with 12% FBS and 1% penicillin/streptomycin, in an atmosphere of 5% CO_2_ and 95% humidity at 37 °C. After growth expanded by 80% of substrate area, subcultivation was performed for plating in T-25 flasks. Adherent cells were dissociated by incubation in 0.1% trypsin at 37 °C for 5 min, and mechanically detached using a cell scraper, followed by 1500 rpm of centrifugation for 5 min to obtain the cell suspension. A vital cell number was determined using a hemocytometer with 0.04% trypan blue staining. The 5,000 cells of each passage were seeded in triplicate into each well of the 96-well plate and incubated with 1 ml Dulbecco’s Modified Eagle Medium (DMEM) supplemented with 10% Fetal Bovine Serum (FBS) for 24 hr. After that, the cells were treated with 1 of 4 conditions: pure media (control), 5 mg/mL D-gal, 100 nM regular insulin, or 5 mg/mL D-gal plus 100 nM regular insulin. This dose of insulin was previously proved to cause insulin resistance in RAW 264.7 cells^66^. After cell treatment in the assigned media, cell viability was measured by MTT assay and expressed as percentage of cell survival relative to the untreated cells. The absorbance to assess cell viability was read at 540 nm using a microplate reader (BioTek, USA).

### Cell differentiation

After seeding for 24 h, cells were plated in triplicate and pre-treated for 30 min with four different conditions as mentioned earlier, followed by a 5-day incubation with 50 ng/mL soluble RANKL (462-TEC, R&D systems, Minneapolis, MN, USA) to induce osteoclastogenesis. Subsequently, cells were fixed in 4% paraformaldehyde for 10 min, and then stained for determination of TRAP activity. A commercial TRAP staining kit (Cosmo Bio, Tokyo, Japan) was used at 37 °C for 60 min to determine osteoclast formation. TRAP-positive multinucleated cells, which had more than 3 nuclei, were considered to denote achievement of osteoclast differentiation. The osteoclast number was finally determined using ImageJ software (NIH, MA, USA).

### Statistical analysis

All data were analyzed using GraphPad Prism version 8.2.1. A one-way and three-way ANOVA with Fisher’s LSD test were performed on the data from the *in vitro* and *in vivo* studies, respectively, to test the difference between groups. Statistical significance was set at a *p*-value of less than 0.05.

## Supplementary Information

Supplementary Information.

## Data Availability

The datasets used and/or analyzed in this study are available from the corresponding author on reasonable request.

## Abbreviations

AGEs: Advanced glycation end products
AUC: Area under the curve
BV: Bone volume
BV/TV: Bone volume fraction or bone volume per tissue volume
CTX-I: C-terminal telopeptide of type I collagen
HFD: High-fat diet
HFDD: High-fat diet with D-galactose
D-gal: D-galactose
HFV: High-fat diet with vehicle
HOMA-IR: Homeostasis model assessment
IL-1β: Interleukin 1 beta
IL-6: Interleukin 6
MDA: Malondialdehyde
NADPH: Nicotinamide adenine dinucleotide phosphate
NDD: Normal diet with D-galactose
NDV: Normal diet with vehicle
OGTT: Oral glucose tolerance test
P1NP: Procollagen type I N-terminal propeptide
RANKL: Receptor activator of nuclear factor κB ligand
ROS: Reactive oxygen species
sRAGE: Soluble receptor for advanced glycation end products
Tb.N: Trabecular number
Tb.Sp: Trabecular separation
Tb.Th: Trabecular thickness
TNF-α: Tumor necrosis factor alpha
TRAP: Tartrate-resistant acid phosphatase
TV: Tissue volume
