# Supplementary material for: D-galactose-induced aging aggravates obesity-induced bone dyshomeostasis
Source: Sci Rep. 2022 May 20;12:8580. doi: 10.1038/s41598-022-12206-4 (PMC9123171; doi:10.1038/s41598-022-12206-4)
Supplement: Supplementary file 1 — Supplementary Information. [file 41598_2022_12206_MOESM1_ESM.docx]

**Supplementary Figures and Table**

**Figure legends**

**Supplementary Fig 1: Correlation between CTX-I and LDL.** (a) in all groups (b) in NDV4, NDV8, HFV4, and HFV8 group. HFDD, high-fat diet with D-galactose; HFV, high-fat diet with vehicle; NDD, normal diet with D-galactose; NDV, normal diet with vehicle.

**Supplementary Fig 2: A summary of *in vitro* and *in vivo* experimental protocols.** AGEs, advanced glycation end products; BV/TV, bone volume per tissue volume; CTX-I, C-terminal telopeptide of type I collagen; HFD, high-fat diet; HFDD, high-fat diet with D-galactose; HFV, high-fat diet with vehicle; MDA, Malondialdehyde; NDD, normal diet with D-galactose ; NDV, normal diet with vehicle; P1NP, procollagen type I N-terminal propeptide; RANKL, receptor activator of nuclear factor κB; sRAGE, soluble receptor for advanced glycation end products; Tb.N, trabecular number; Tb.Sp, trabecular separation; Tb.Th, trabecular thickness.

**Supplementary Fig. 3** The Western blot bands without being cropped showing the molecular weight of soluble receptor of advanced glycation end-products (sRAGE) in serum (n = 6/group).

**Supplementary Fig. 4** The Western blot bands without being cropped showing the molecular weight of transferrin in serum (n = 6/group).


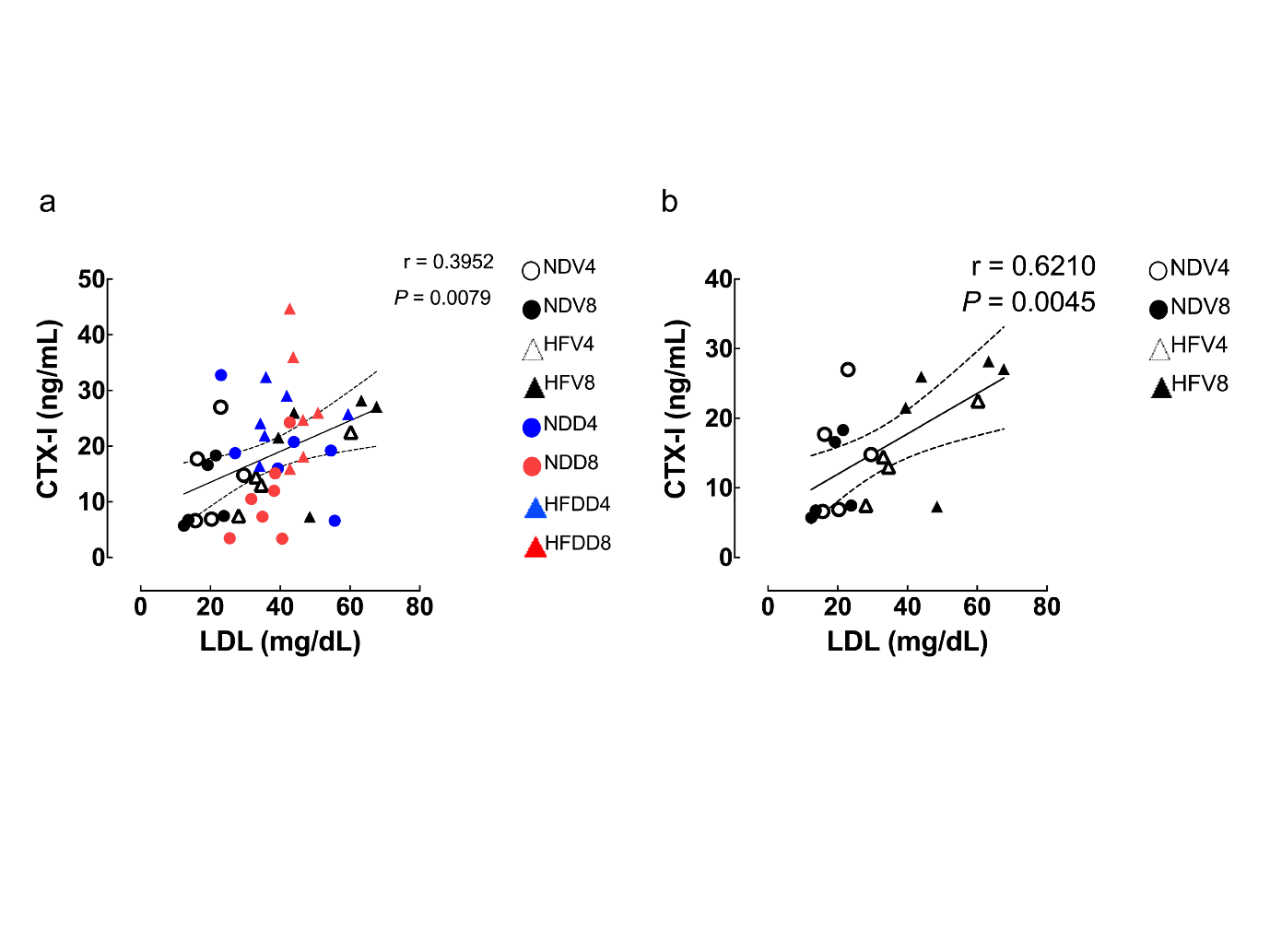


**Supplementary Fig. 1**

**
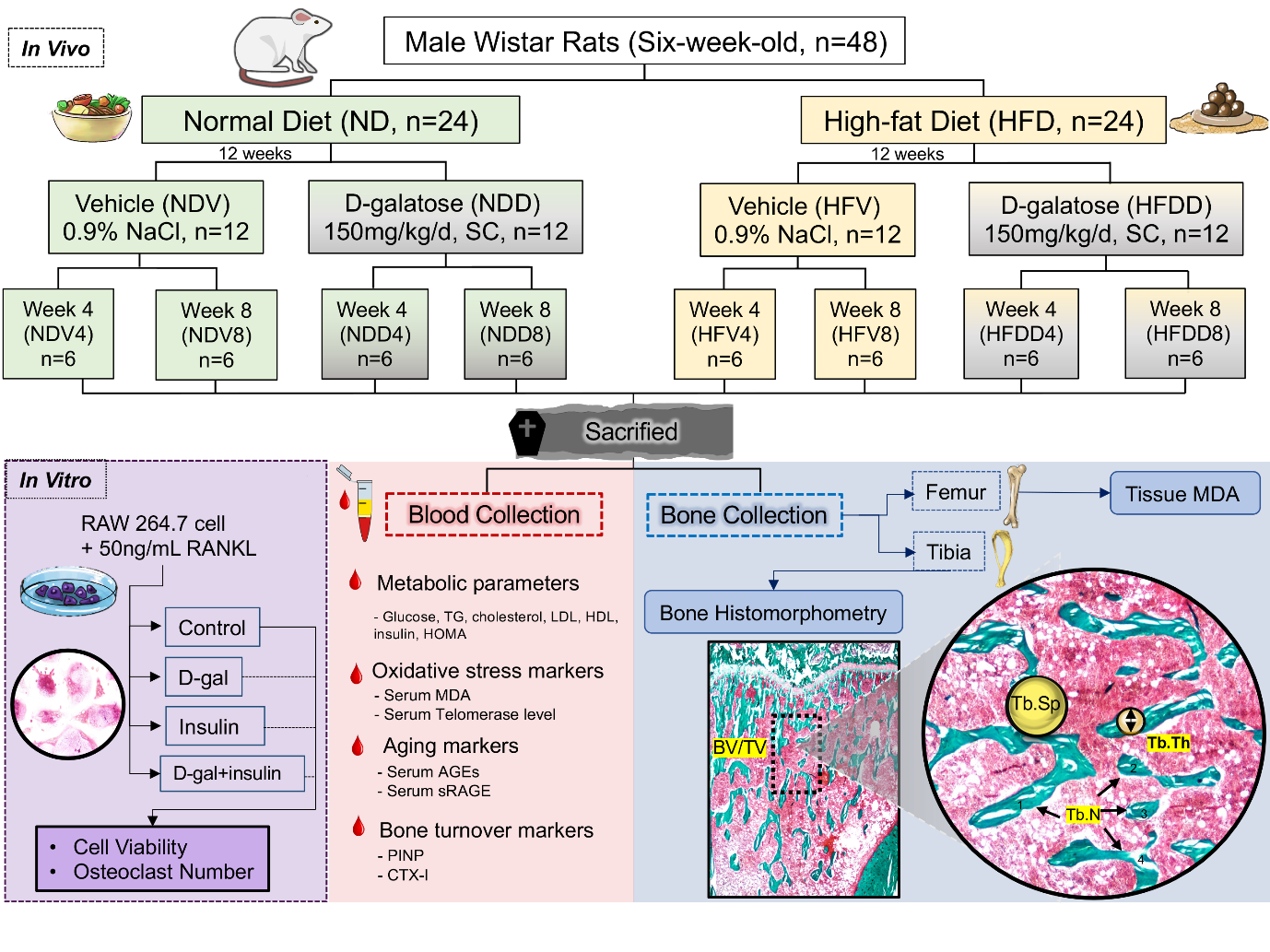
**

**Supplementary Fig. 2**

**
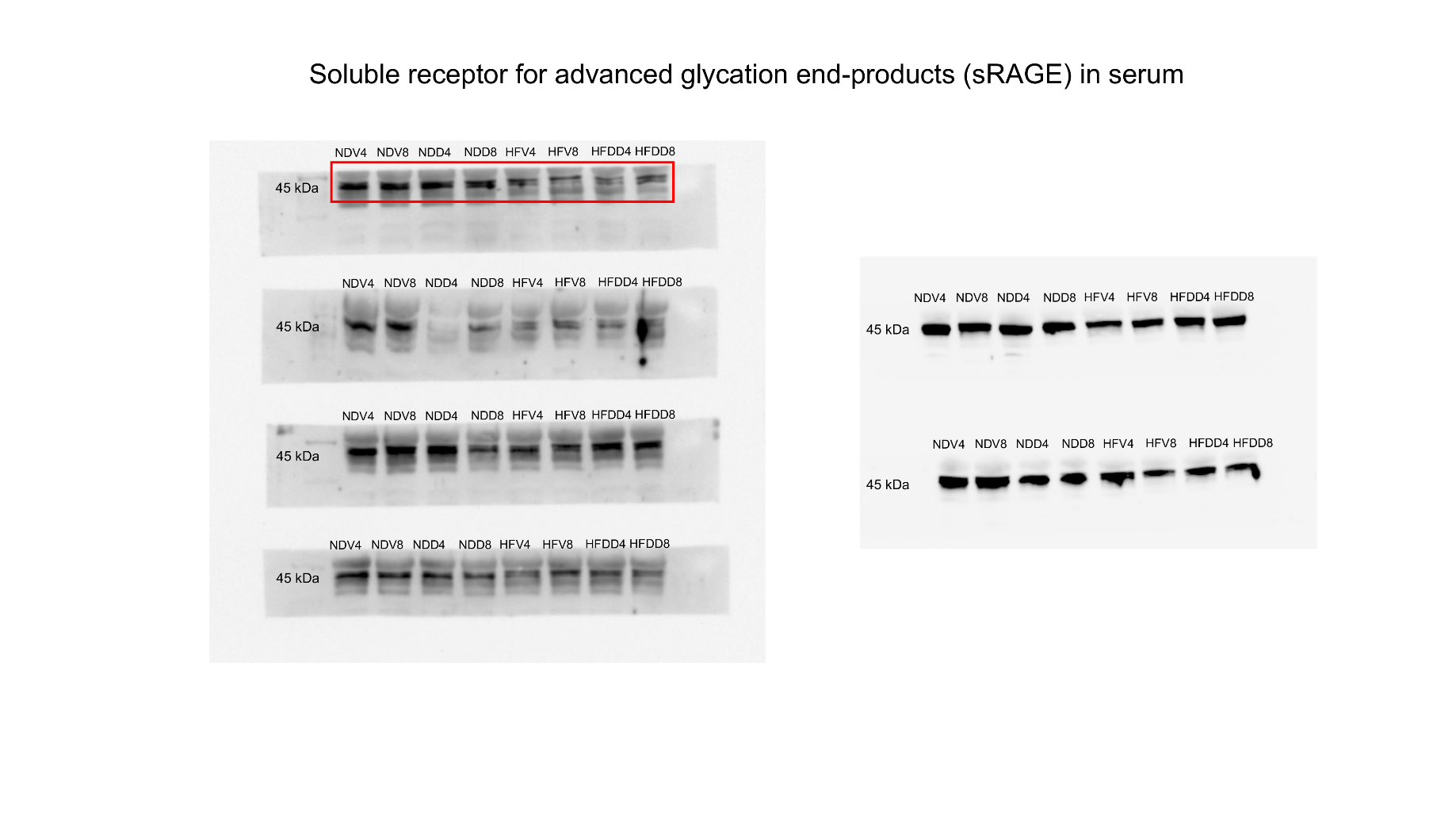
**

**Supplementary Fig. 3**

**
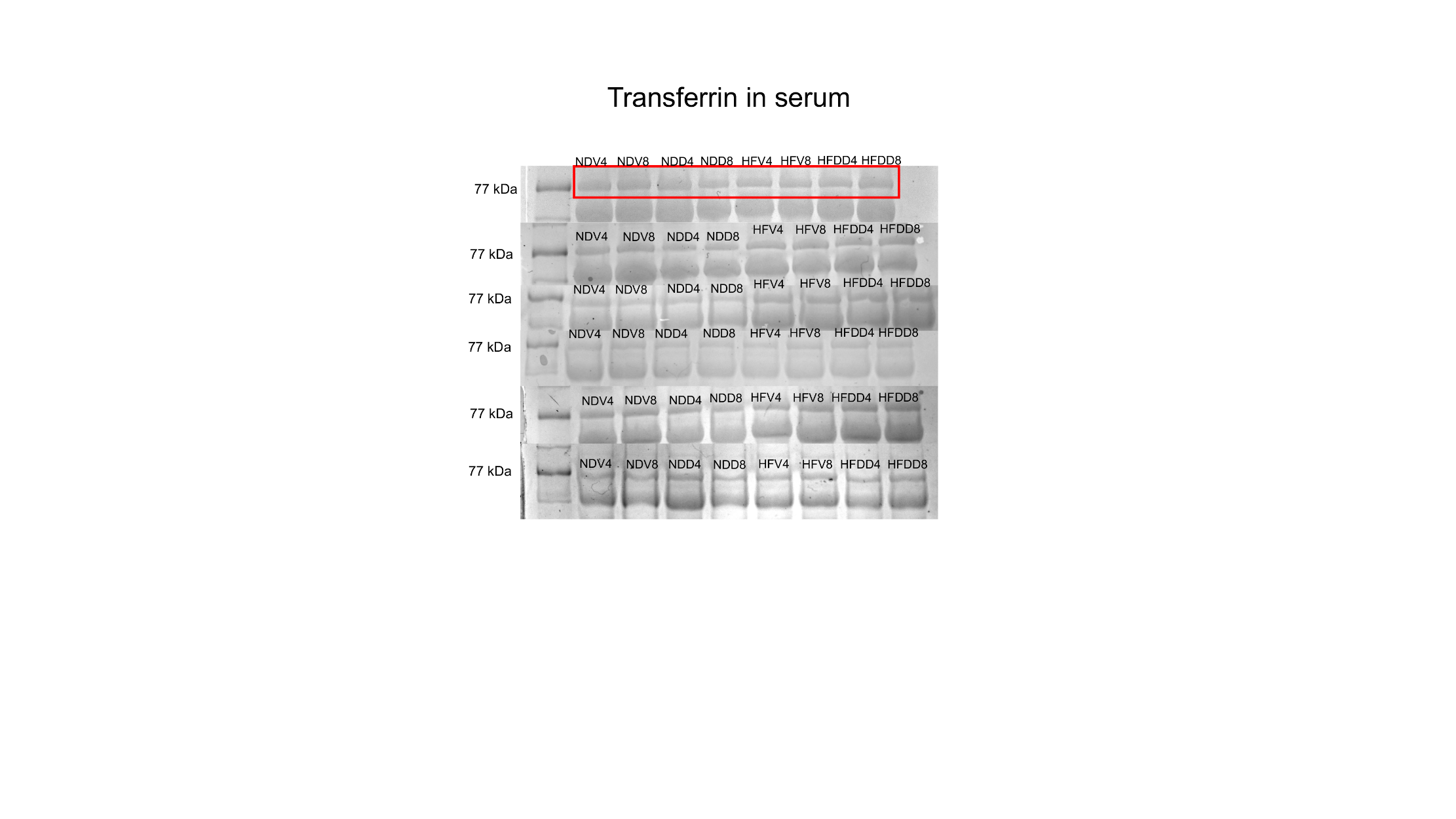
**

**Supplementary Fig. 4**

**Supplementary Table 1:** List of primers used for qRT-PCR

| Gene | Primer sequences (5’ to 3’) |  |
| --- | --- | --- |
| RANKL | Forward: GAC AGG CAC GGA CTC GTA  Reverse: CGC TCA TGC TAG TCG TCT A |  |
| TNF-α | Forward: GCT GCA CTT TGG AGT GAT CG  Reverse: CTT ACC TAC AAC ATG GGC TAC AG | |
| IL1- β | Forward: CAC CTC TCA AGC AGA GCA CAG  Reverse: GGG TTC CAT GGT GAA GTC AAC | |
| IL-6 | Forward: ATG AAC TCC TTC TCC ACA AGC GC  Reverse: GAA GAG CCC TCA GGC TGG ACT G |  |
| β-actin | Forward: GGAGATTACTGCCCTGGCTCCTA  Reverse: GACTCATCGTACTCCTGCTTGCTG |  |
